# Case Report: Variegate porphyria disclosed by post-gastric bypass complications and causing predominant painful sensorimotor axonal peripheral neuropathy

**DOI:** 10.3389/fgene.2022.993453

**Published:** 2022-10-31

**Authors:** Edwige Collaud, Luis Wittwer, Anna-Elisabeth Minder, Jean-Marie Annoni, Elisabeth I. Minder, Joelle N. Chabwine

**Affiliations:** ^1^ Division of Neurorehabilitation, Fribourg Hospital Meyriez/Murten, Fribourg, Switzerland; ^2^ Division of Medicine, Emmental Hospital, Burgdorf, Switzerland; ^3^ Division of Endocrinology, Diabetology, Porphyria, Triemli Hospital, Zurich, Switzerland; ^4^ Neurology Unit, Laboratory for Cognitive and Neurological Science, Department of Neuroscience and Movement Science, Faculty of Science and Medicine, University of Fribourg, Fribourg, Switzerland

**Keywords:** variegate porphyria, pain, polyneuropathy, gastric bypass, bariatric surgery, PPOX mutation

## Abstract

**Background and aims:** Porphyrias constitute a group of rare genetic diseases due to various, mostly autosomal dominant mutations, causing enzymatic deficiency in heme biosynthesis. As a result, neurotoxic porphyrin precursors and light-sensitive porphyrins accumulate, while dysfunction in their targets determines the disease symptoms. Variegate porphyria (VP), one of the acute hepatic porphyrias, is caused by a protoporphyrinogen oxidase (PPOX) mutation. During acute attacks, among other factors, triggered by drugs, stressors, or fasting, an increase in urinary and fecal porphobilinogen (PBG), aminolevulinic acid (ALA), and porphyrins occurs, damaging the autonomous, peripheral, or central nervous system. The disease remains often latent or displays minimal symptoms usually overlooked, exposing undiagnosed patients to potentially serious complications in the presence of the aforementioned triggers.

**Case report:** This 46-year-old woman presented, some days after a bariatric surgery, with severe flaccid tetraparesis and neuropathic pain, initially misdiagnosed as a functional neurological disorder. The severe axonal sensorimotor polyneuropathy led to further investigations, disclosing high urinary porphobilinogen, ALA, and porphyrin levels due to a new PPOX mutation. Retrospectively, it appeared that the patient had had typical VP symptoms (abdominal pain, fragile skin, and dark urine episodes) for years prior to the surgery. Treated with carbohydrate load, neurorehabilitation, and analgesics, she slowly recovered to full mobility, with partial autonomy in her daily life activities, although fatigue and severe pain persisted, preventing her from returning to work.

**Conclusion:** This case documents gastric bypass surgery as a trigger of severe VP invalidating neurological symptoms and illustrates how the delayed diagnosis and post-interventional complications could have been prevented by screening for porphyria cardinal symptoms prior to the intervention. Likewise, this cost-effective screening should be performed before any treatment influencing the diet, which would dramatically improve the porphyria diagnosis rate and outcome.

## Background

Porphyrias are a group of rare genetic diseases, caused by a deficiency in one of the eight enzymes of heme biosynthesis, which results in the accumulation of porphyrin precursors (Kauppinen, 2005). In autosomal dominant acute porphyrias, porphyrin precursors, in particular aminolevulinic acid (ALA), act toxic to the central, peripheral, and autonomic nervous system, causing acute abdominal pain and neuropathy in the context of an acute porphyria attack (Phillips, 2019). In addition to the most prevalent form of acute porphyria, the acute intermittent porphyria (AIP) variegate porphyria (VP) constitutes an autosomal dominant inherited form of porphyria caused by a mutation in the protoporphyrinogen oxidase (PPOX) gene (Tabaro et al., 2018). The latter results in the accumulation of toxic porphyrin precursors due to enzyme deficiency, especially provoking acute neurovisceral and cutaneous symptoms. The exact prevalence of VP mutations is unknown due to their low penetrance and most probably because porphyria symptoms are often overlooked (Chen et al., 2016).

Acute porphyria attacks consist of acute neurovisceral pain without peritonism, associated with pre-ileus, constipation, and symptoms of dysautonomia (tachycardia, hypertension, nausea, and vomiting). Hyponatremia, sensorimotor polyneuropathic symptoms, and red or dark urine episodes can also occur (Sassa, 2006). The acute porphyria attack is confirmed by the elevation of porphyrin precursor excretion in the urine, namely, porphobilinogen (PBG) and ALA (Pischik and Kauppinen, 2015). The most frequent triggers of a porphyria attack are various drugs, surgical procedures, fasting, smoking, and physical or psychological stressors (Sassa, 2006; Zürich and Endokrinologie, 2018). Severe porphyria attacks can be life-threatening or result in irreversible damage, with persistent disability due to neurological deficits (Sassa, 2006). The treatment of acute porphyria attacks consists in reducing the accumulation of toxic compounds by intravenous infusion of glucose and heme inhibiting D-ALA-synthase 1. This treatment consistently prevents persistent disabilities, while eviction of triggering factors allows for avoiding porphyria attacks (Pischik and Kauppinen, 2015). Thus, porphyria can be adequately managed through well-established guidelines, under the condition that it is diagnosed.

We report in this study a patient who abruptly developed a painful flaccid tetraparesis, following gastric bypass surgery. The initial wrong diagnosis of a functional neurological disorder delayed the diagnosis of VP for several months, condemning the patient to long-term neurological complications.

## Case report

This 46-year-old woman known for obesity class III (BMI 41.7kg/m^2^) underwent a gastric bypass surgery 2 months before admission to the rehabilitation clinic. Her medical history was otherwise remarkable for depression in a burn-out context 4 years earlier, smoking (25 p.y.), and non-specific chronic low back pain. Immediately after the gastric bypass, she displayed an abdominal wall hernia and developed severe complications, interpreted as anastomosis insufficiency, gastric ulcer, or wound abscess, leading to new operations and a prolonged hospital stay. Two weeks after discharge, she was re-admitted to the emergency department for an acute onset tetraparesis. A first diagnostic workup including a normal spinal cord MRI was inconclusive, and with respect to her prior history of depression, the tetraparesis was labeled as a functional neurological disorder. Subsequently, the patient was referred to our hospital for neurorehabilitation.

On arrival, she complained of severe fatigue, bilateral limb weakness, hypoesthesia, and pain, dysgeusia for all tastes, and dysphagia. A neurological examination disclosed bilateral facial peripheral paresis, proximally pronounced severe flaccid tetraparesis with tendon hyporeflexia, bilateral touch and pick hypoesthesia in the trigeminal area, and C1 dermatomes, as well as a glove and stocking distal hypoesthesia. The sensory abnormalities fluctuated between repeated examinations. The neuropsychological evaluation revealed no cognitive impairment, while the patient displayed anxiety and depression symptoms in response to her severe disability and the general health situation.

## Diagnostic procedures

A new MRI confirmed normal findings in the brain and the spinal cord, as well as in the proximal portion of spinal nerve roots. The peripheral pattern of neurological symptoms warranted electroneuromyography, which confirmed axonal sensorimotor neuropathy involving all four limbs. A broad etiological search (Table 1, supplementary material) ruled out infectious diseases (normal serologies for syphilis, human immunodeficiency virus (HIV), cytomegalovirus (CMV), Epstein-Barr virus (EBV), and *Borrelia burgdorferi*, as well as the negative cerebrospinal fluid (CSF) polymerase chain reaction (PCR) for CMV and EBV), endocrine disorders (diabetes and thyroid dysfunction), and Wilson’s disease (serum and urine copper measurements). No relevant vitamin deficiency explaining the neurological findings was detected, except for low levels of vitamin D [22 nmol/l] and folate [3.4 nmol/l]) (Punchai et al., 2017). There were signs of systemic inflammation with an increased blood erythrocyte sedimentation rate (BSR) [50 mm/h], elevated serum C3c, and 8.9% of monocytes in the white blood count. Local production of oligoclonal bands in the CSF was excluded. Autoimmune disease screening identified an anti-amphiphysin antibody in the serum (no titer mentioned).

**TABLE 1:** Investigations over time.

Date	Investigation	Result
Initial evaluations and investigations		
22.04.18	Total column MRI	Normal
09.05.18	Test to acetylcholinesterase inhibitors (pyridostigmine 60 mg)	Negative
11.05.18	ENMG	Signs of axonal neuropathy in the four limbs, with acute and chronic components; secondary muscular abnormalities not excluded
15.05.18	Brain MRI	Normal
16.05.18	CSF examinations	Normal white cell count, normal protein, normal immunologic electrophoresis, and absence of local production of oligoclonal bands
16.05.18	Blood examinations	**Positive DOT for antineuronal antibodies (anti-amphiphysin)/elevated BSR (50 mm/h)/light increase of the C3 complement (1.43 g/L)/increased monocyte rate (8.9%)**
		Negative DOT for screening of autoimmune diseases (including myasthenia gravis antibodies)
		Negative screening for the most frequent neuro-infectious diseases (syphilis, HIV, Lyme, CMV, and EBV)
		Normal vitamins B1, B6, B12, B9, D, E, and A, but hypovitaminosis D
		Absence of metabolic diseases (normal HbA1c and TSH) and normal metal dosage (copper and zinc)
		**Increased urinary porphyrins and porphyrin precursors**
01.07.18	Neuropsychological evaluation	Normal to slightly diminished performance in the executive functions and working memory
Second-line/follow-up evaluations and investigations		
10.07.18	Laboratory examinations	Negative DOT antineuronal antibody in the serum and in the CSF/mild BSR increase (13 mm/h)/normal C3 complement, CRP, and monocytes
12.07.18	ENMG (evolution 1)	Stable (no change from the initial testing)
17.07.18	Genetic testing (PPOX gene mutation)	**Mutation of protoporphyrinogen oxidase (exon 4 of the PPOX gene)**
17.09.19	ENMG (evolution 2)	Improvement of the nerve conduction velocity of the right peroneus nerve, focal myelopathy of the right median nerve suggesting a carpal tunnel syndrome, and late spinal response but not prolonged right tibial median nerves
26.11.19	MOCA score	25/30
26.11.19	Neuropsychological evaluation	Normal executive functions and working memory, some attention difficulties (treatment velocity) attributed to the newly diagnosed genetic disease and chronic pain; no other cognitive dysfunction

Important positive results are written in bold.

In the meantime, second-line complementary results disclosed high porphyrin precursors and porphyrin levels in the urine, with a positive plasmafluorescence scan [626 nm], as well as elevated coproporphyrin I [46.3 nmol/g] and III [350 nmol/g], and protoporphyrin [1444 nmol/g]. Altogether, these findings, in addition to the above-detailed clinical picture, confirmed the diagnosis of VP (Hift et al., 2004) (Table 2, supplementary material). Moreover, the patient exhibited a heterozygous deletion in exon 4 of the PPOX gene (Table 1, supplementary material). To the best of our knowledge, this mutation has never been reported before.

**TABLE 2:** Value of porphyrins over time with references.

	Measured porphyrin(s)	[Urinary porphyrins/creatinine] Reference values^a^		[Porphyrins/24h]^b^		[Feces]^c^	
28.05.18	Total porphyrins	902	[<30]	4606	[<310]		
	ALA	18.7	[<2.5]				
	Porphobilinogen	2.63	[<1.25]				
	Uroporphyrin	35.7	[<4.6]				
	Heptacarboxyporphyrin	48.7	[<0.8]				
	Hexacarboxyporphyrin	29.1	[<2.2]				
	Pentacarboxyporphyrin	145	[<1.6]				
	Coproporphyrin I	30.7	[<5.8]				
	Coproporphyrin III	553	[<21]				
05.06.18	PBG-deaminase			136	[9]		
	Plasmafluorescence scan			Peak maximum at 626 nm			
	Coproporphyrin I					46.3	[<20]
	Coproporphyrin III					350	[<12]
	Protoporphyrin					1444	[<80]
20.06.18	Total porphyrins	126.6	[<30]	884	[<310]		
	ALA	5.3	[<2.5]	37.1	[<50]		
	Porphobilinogen	1.12	[<1.25]	7.8	[<7.5]		
	Uroporphyrin	29.1	[<4.6]	203.3	[<60]		
	Heptacarboxyporphyrin	8.9	[<0.8]	61.9	[<10]		
	Hexacarboxyporphyrin	1.5	[<2.2]	10.5	[<21]		
	Pentacarboxyporphyrin	7.2	[<1.6]	50.4	[<16]		
	Coproporphyrin I	9.8	[<5.8]	68.2	[<100]		
	Coproporphyrin III	70.2	[<21]	430	[<210]		
28.09.18	Total porphyrins	60	[<30]				
	ALA	6.9	[<2.5]				
	Porphobilinogen	0.52	[<1.25]				
	Uroporphyrin	18.2	[<4.6]				
	Heptacarboxyporphyrin	5.3	[<0.8]				
	Hexacarboxyporphyrin	0.7	[<2.2]				
	Pentacarboxyporphyrin	7.2	[<1.6]				
	Coproporphyrin I	6.6	[<5.8]				
	Coproporphyrin III	42	[<21]				
16.01.19	Total porphyrins	133	[<30]				
	ALA	7.3	[<2.5]				
	Porphobilinogen	5	[<1.25]				
	Uroporphyrin	24.6	[<4.6]				
	Heptacarboxyporphyrin	5.8	[<0.8]				
	Hexacarboxyporphyrin	1.3	[<2.2]				
	Pentacarboxyporphyrin	9.1	[<1.6]				
	Coproporphyrin I	5.8	[<5.8]				
	Coproporphyrin III	86	[<21]				
09.03.19	Total porphyrins	189	[<30]				
	ALA	8,3	[<2.5]				
	Porphobilinogen	0.63	[<1.25]				
	Uroporphyrin	30.6	[<4.6]				
	Heptacarboxyporphyrin	8.3	[<0.8]				
	Hexacarboxyporphyrin	2.7	[<2.2]				
	Pentacarboxyporphyrin	19.2	[<1.6]				
	Coproporphyrin I	10.6	[<5.8]				
	Coproporphyrin III	117	[<21]				
17.06.19	Porphobilinogen	2.49	[<1.25]				
12.12.19	ALA	8.3	[<2.5]				
	Porphobilinogen	3.75	[<1.25]				

^a^ Aminolevulinic acid, porphobilinogen in µmol/mmol, and urinary porphyrins in µmol/mol.

^b^ Aminolevulinic acid, porphobilinogen in µmol/24h, and urinary porphyrins in nmol/24h.

^c^ In nmol/g dry weight.

When further retrospectively questioning the patient and more accurately examining her, it appeared that she had thin and fragile skin (prevailing as far back as she could remember in her past), however, without the typical blistering seen in VP. In addition, she repeatedly observed that her urines were dark-colored. Otherwise, she was treated for irritable bowel syndrome (metixen, pepsine, dimeticon, glutaminic acid hydrochloride, pancreatin, cellulose, and dehydrocholic acid) prior to the bariatric surgery. Her family history was unremarkable for diagnosed porphyria or evoking symptoms.

## Management and follow-up

Due to the positive serum anti-amphiphysin antibody, an autoimmune origin of polyneuropathy was initially suspected, indicating an oral steroid therapy course for few weeks. The patient’s mobility, dysphagia, and sensory complaints progressively improved, without further aggravation at the end of the treatment, while the anti-amphiphysin antibody and inflammation signs disappeared in the serum.

**FIGURE 1:**
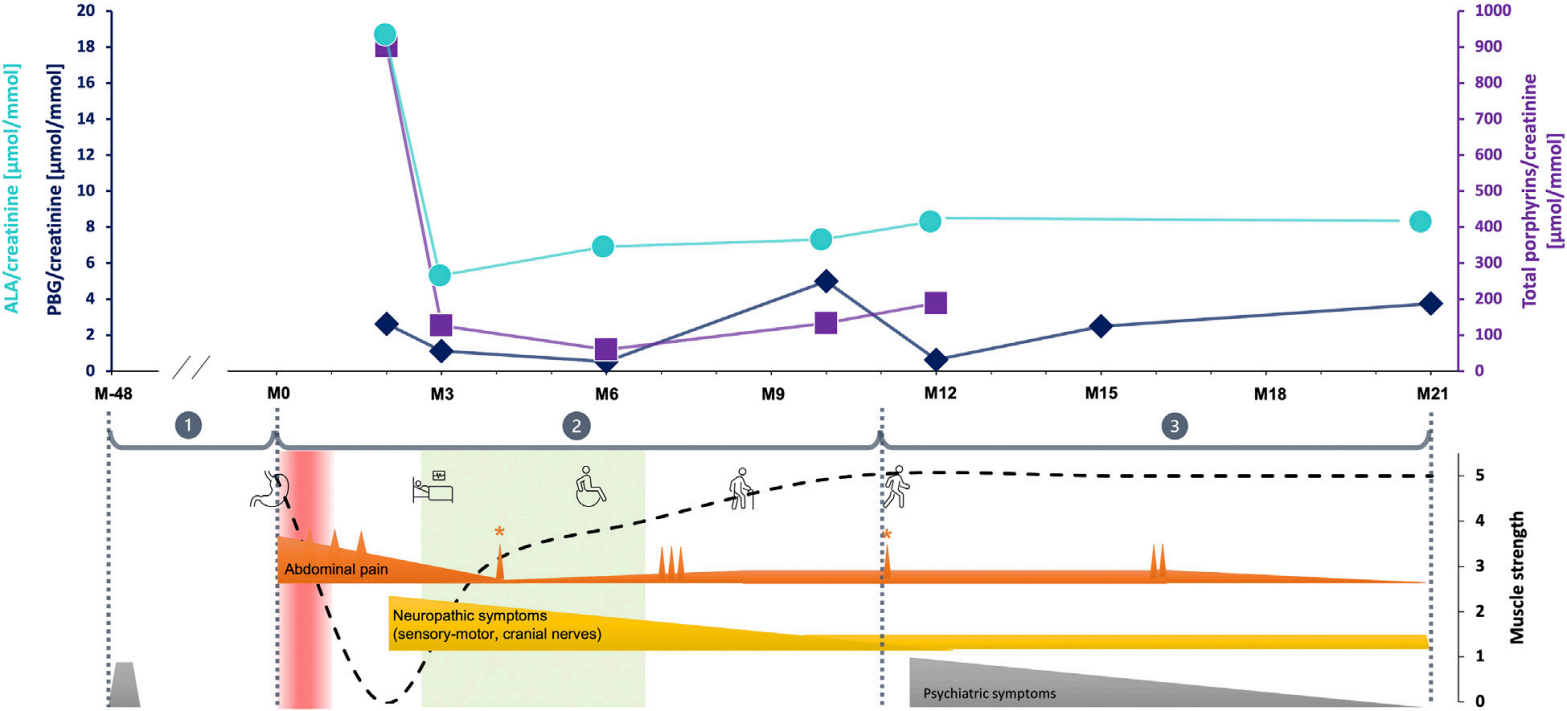
The evolution of clinical symptoms and urinary porphyrins through the follow up. The vertical red band labels the acute abdominal pain period supposedly corresponding the acute porphyria attack, while the light green is related to the stationary neurorehabilitation. The numbers within the grey circles represent the key observation periods corresponding the main evolutionary/intervention steps, as following (M month, the digit following M represents the number of months before or after the gastric bypass procedure): ① [M-48 until 01.2018 (M0)] is the retrospective period; ② [from M0 to M11 (01.2019)] corresponds to the period during which the main and most severe symptoms as well as therapeutic interventions in hospitals occurred; ③ [from M11 until the end of the follow up at M21 (12.2019)] represents the post-stationary evolution. Urinary porphyrins concentrations are labelled as following: turquoise blue dots for ALA, dark blue rhombs for PBG and violet squares for total porphyrins. Main clinical symptoms evolution over the observation period. Ordinate values are arbitrary units corresponding to a clinical estimate of symptom severity. The main symptoms were grouped in three categories represented by three different color codes: abdominal pain in orange, peripheral neuropathic signs in yellow and psychiatric symptoms in grey. The functional performance and main interventions are shown by illustrative cartoons: the stomach for bariatric surgery, the bed for the period when the patient was bed-ridden, the wheelchair and walking stick and free walking showing the improving patterns of mobility. ① burn-out 2014 (grey pyramid); ② acute abdominal pain with maximal intensity during the period considered to correspond to the porphyria attack, with small peaks occurring at surgical interventions (small orange spines above the baseline). The pain intensity decreased as rehabilitation and other treatments were initiated, and stabilized at very low level although 3 repetitive peaks took place at M7-M8. Of notice, two isolated peaks were recorded during appendagitis exacerbation (with * above) at M4 and M11. Neuropathic symptoms started later than abdominal pain as characteristic for porphyria. They were at their maximum from the beginning, but progressively decreased during neurorehabilitation program parallel to the overall clinical state of the patient. They finally dropped to a low, but persistent steady-state level; ③ during this post-stationary period, both abdominal pain and neuropathic symptoms remained low (except two exacerbations of abdominal pain at M16). Psychiatric symptoms including sleep disorders, fatigue, anxiety and depression reappeared, but progressively improved with appropriate medication.

After the diagnosis of VP, the therapeutic approach was to the increase carbohydrate intake to a minimum of 250 g/day, as well as the eviction of all porphyrinogenic drugs (such as fentanyl) and other potential acute attack-triggering factors. Because the patient was not considered to be suffering an acute attack, no heme arginate treatment was applied. An intensive multidisciplinary neurorehabilitation regime and optimal symptom treatments allowed the patient to progressively gain her mobility back, with the improvement of her muscle strength, pain, somatosensory complaints, dysphagia, and dysgeusia (Figure 1). Neuropathic pain and other sensory complaints remained moderately stable despite consistent analgesic therapy (fentanyl patch replaced by tapentadol, pregabalin, and amitriptyline), while mood disorders and insomnia improved, thanks to mirtazapine. However, she developed acute abdominal pain during her neurorehabilitation stay due to appendagitis, efficiently treated by appropriate analgesia.

She was discharged after 6 weeks of stationary neurorehabilitation and continued the ambulant physical and occupational therapy, with further functional improvement (Table 3, supplementary material). After the surgical cure of abdominal hernia and appendagitis healing, abdominal pain disappeared. However, severe fatigue persisted in addition to limb neuropathic pain and preexisting low back pain, preventing the patient from returning to work. Nevertheless, she was able to care for her children and perform her daily household tasks. Family genetic counseling was carried out as proposed in porphyria guidelines (Anderson et al., 2005).

**TABLE 3:** Symptoms and clinical signs over time.

Date	Subjective symptom	Objective sign
12.02.18 (M0)	Brutal abdominal pain	Mimicry acute abdomen
05.04.18	Diminished general state, asthenia, inappetence, and abdominal pain with belt-knife knock	Weak patellar reflexes, Mingazzini hardly testable, loss of superficial sensitivity discrimination to « pick and touch» and proprioception, walking becoming impossible, sphincter tone diminished, impossibility to whistle, and suboptimal cheek blowing
	Pelvis paresthesia, fecal incontinence, hematuric urine, and weakness of the low limbs from distal to proximal and then upper limbs from proximal to distal	
30.04.18	Bilateral loss of face sensitivity	Proximal predominant tetraparesis
	Dysphagia	Weak reflexes
	Weakness and pain in limbs	Hypoesthesia without a precise dermatome DD trigeminal nerve and C1 region
	Balance and body scheme disorder (probably due to deconditioning)	Hypoesthesia to pick and touch the distal upper and lower limbs
		Simpson’s test: seems positive
		Pigmented skin lesion behind the hands
20.06.18 (M4)	Inconclusive abdominal pain	
28.09.18 (M7)	Abdominal pain independently from eating 3–4 times a week	Strength and sensitivity improvement
	Bad healing, skin fragility (behind the hands and face) and face hirsutismus	
16.01.19 (M11)	Low belly pain with bloating, neuropathic pain of lower limbs and body, less general strength, fatigue, skin fragility, appendagitis, and neurologic recovery	Walking without help inside and with a walking stick outside
18.02.19	Neuropathic pain, insomnia, anxiety (due to pain and hospitalization’s context), and tremor	
23.05.20	Electric discharge in all limbs and face and sensation recovery until elbows and knees	More expressive facial gestures and complete eye closing
17.06.19 (M16)	Abdominal pain for 3 h every 2 days, especially in the evening, and sometimes stomach burning	Strength of lower limbs is still reduced but better than last time
	Reduction of sensation and strength	
	Neuropathic pain waking up at night	
01.07.19		Sensory affect (superficial, paresthesia, and deep pain) and motor impairment of 4 limbs predominant to the LL and diminished impairment of bilateral cranial nerves V and VII
17.09.19	Electric discharge in all limbs and muscle fatigability	Bilateral facial paresis predominant to the right side, suboptimal whistle and cheek blowing, bilateral facial paresthesia (predominant right), weak Souques’ sign right, weak reflexes of 4 limbs, pallesthesia 6/8 at fingers, 5/8 at toes, and light trunk instability at Romberg testing
29.11.19	Stability of pain and sensitivity (UL: elbows; LL: feet), without exacerbation, and insomnia	Stable neurologic state and slightly clinical improving
12.12.19 (M21)	Total body electric pain, in the evening, caused by physical activity, fatigue, walking for 200–300 m, and sleep disorder	

## Discussion

A few weeks after undergoing a gastric bypass procedure with several postoperative complications, this middle-aged patient developed an abrupt proximal-predominant areflexia and flaccid tetraparesis associated with sensory symptoms (including neuropathic pain) involving the four limbs. After a thorough and stepwise workup excluding etiologies, her neurological symptoms were most likely caused by VP, based on typical abnormalities of urinary and fecal biomarkers (Di Pierro et al., 2021) and the finding of a new heterozygous mutation in the PPOX gene of pathological potential never reported before. Indeed, being a deletion, the observed mutation caused a frameshift and a truncated transcript most likely leading to the synthesis of a dysfunctional enzyme and subsequent toxic porphyrin precursors (Barbaro et al., 2013). The unexpected discrepancy between urinary ALA and PBG increases could be explained by their biological variations (Floderus et al., 2006) and possible differences in assay specificities (Agarwal et al., 2021), while positive plasma fluorescence and the ratio coproporphyrin III: I of ∼8 further confirmed the diagnosis of porphyria. Despite the high prevalence reported in South Africa, VP is relatively frequent in Switzerland due to founder mutations (Schneider-Yin and Minder, 2006). Whether the patient was still undergoing a porphyria attack at the diagnosis time is an interesting question. The modest increase of urinary ALA alone (∼7 times the reference values) and the absence of dark-colored urines were against this hypothesis. However, taking into account the decreasing kinetics of urinary markers and further stabilization thereafter, the patient’s early postoperative abdominal pain episode and acute onset of sensorimotor deficits might have been caused by an acute porphyria attack, the latter being triggered by the bariatric surgery procedure.

The role of gastric bypass in triggering (or exacerbating latent) VP symptoms is also worthy of discussion. In fact, bariatric surgery encompasses several porphyria-precipitating factors (Anderson et al., 2005). First, exposure to various potentially porphyrinogenic drugs during anesthesia and the postoperative course are considered. Fentanyl prescription and its long maintenance should have deserved great caution in this patient. Not only this opiate is listed among porphyrinogenic drugs (Zürich and Endokrinologie, 2018; N APOS, 2007; Koffman et al., 2006) and could have, as such, contributed to the occurrence of porphyria symptoms but also its chronic use can induce hyperalgesia, aggravating instead of treating the patient’s pain (Roeckel et al., 2016). Additionally, the combination of physical and psychological stress and a long-lasting reduction of caloric (especially carbohydrate) intake can be listed. Furthermore, in individuals with a porphyria mutation, the desired weight loss and subsequent impairment of energy metabolism in the liver trigger the metabolic cascade that results in the accumulation of neurotoxic porphyrin precursors (Danion et al., 2012). At last, the gastric bypass causes hormonal dysfunction, including insulin dysregulation (Koffman et al., 2006; Kumar, 2007; Abdelaal et al., 2017), adding up to homeostasis disruptions. Thus, the gastric bypass context cumulates risks of precipitating a latent (or poorly symptomatic) porphyria to become highly symptomatic, especially if we consider the low penetrance of related mutations (Chen et al., 2016). On the other hand, the few reports in the recent literature probably do not reflect the real (higher) incidence of such cases, of which careful documentation and reporting should raise awareness among professionals and avoid unnecessary diagnosis delay, as in the case reported in this study.

The presence of anti-amphiphysin antibodies initially questioned an inflammatory background to the axonal polyneuropathy (Dubey et al., 2019) and/or a link with the ongoing porphyria. On one hand, axonal polyneuropathy is not the most typical neurological manifestation associated with the presence of these antibodies (Moon et al., 2014), and no relapse was noticed after definitely stopping steroids. On the other hand, previous reports of concomitant AIP and autoimmune disease (e.g., systemic lupus erythematosus and Sjögren syndrome) consider the latter (or their treatments) as a precipitating rather than a causal factor (Esteve-Valverde et al., 2020; Teng et al., 2020), although there are indications that AIP could be accompanied by systemic inflammatory, but non-autoimmune, reactions (Storjord et al., 2017). Taking into account the absence of a plausible causal relationship between anti-amphiphysin antibodies and both the patient’s polyneuropathy and porphyria, we finally hypothesized that the transient systemic inflammatory reaction was non-specific and consecutive to repeated surgery (with a crossed reaction to the amphiphysin antigen).

## Conclusion

Overall, VP unequivocally appears to be the only possible etiology of this patient’s neurological symptoms, with supporting clinical, biological, and genetic evidence. Detailed and oriented medical history revealed that she had, before undergoing bariatric surgery, symptoms (intermittently dark reddish urine and fragility of the skin when exposed to the light) that could have, if screened for, prompted the search of porphyria (by urinary ALA and PBG measurements) prior to the gastric bypass procedure. Severe abdominal crises without evident cause during the early postoperative period would have been one additional red flag justifying this investigation. The diagnosis of VP enabled the instauration of specific management measures (Anderson et al., 2005) including carbohydrate-based diet, neurorehabilitation, and optimal symptom management, which allowed significant clinical improvement up to a relative autonomy in the activities of daily life, almost a year later. Nevertheless, due to the delayed diagnostic workup and treatment onset, persisting severe fatigue and limb neuropathic pain, despite heavy analgesia, prevented the patient from returning to work.

Considering the increasing prevalence of obesity (Kumar, 2007), which already constitutes a major public health problem (Koffman et al., 2006; Abdelaal et al., 2017), the indication for bariatric surgery (one of the most frequently used therapeutic procedures) will likely raise, exposing more patients with latent porphyria to develop the disease symptoms and potentially serious complications. Therefore, learning lessons from this case, we propose a pre-operative cost-effective screening for symptoms highly evocative of porphyria, especially in obese patients intending to opt for or awaiting gastric bypass. Positive screening would prompt appropriate and timely preventive and therapeutic measures, which efficiently reduce short- and long-term adverse outcomes (Sassa, 2006).

## Data Availability

The original contributions presented in the study are included in the article/Supplementary Material; further inquiries can be directed to the corresponding author.

## Abbreviations

AIP, acute intermittent porphyria; ALA, aminolevulinic acid; BSR, blood sedimentation rate; CMV, cytomegalovirus; CSF, cerebrospinal fluid; EBV, Epstein-Barr virus; HIV, human immunodeficiency virus; PBG, porphobilinogen; PCR, polymerase chain reaction; PPOX, protoporphyrinogen oxidase; VP, porphyria variegate.
